# Where are People Dying in Disasters, and Where is it Being Studied? A Mapping Review of Scientific Articles on Tropical Cyclone Mortality in English and Chinese

**DOI:** 10.1017/S1049023X22000541

**Published:** 2022-06

**Authors:** Caleb Dresser, Alexander Hart, Alex Kwok-Keung Law, Grace Yen Yen Poon, Gregory Ciottone, Satchit Balsari

**Affiliations:** 1. Department of Emergency Medicine, Beth Israel Deaconess Medical Center and Harvard Medical School, Boston, Massachusetts, USA; 2. Assistant Professor, University of Connecticut School of Medicine, Farmington, Connecticut, USA; Director of Research, Disaster Medicine Fellowship, Beth Israel Deaconess Medical Center, Boston, Massachusetts, USA; 3. Accident and Emergency Medicine Academic Unit, The Chinese University of Hong Kong, Hong Kong, China; 4. Director, Disaster Medicine Fellowship, Department of Emergency Medicine, Beth Israel Deaconess Medical Center, Boston, Massachusetts, USA; 5. Department of Global Health and Population, Harvard TH Chan School of Public Health, Boston, Massachusetts, USA

**Keywords:** cyclonic storms, disaster medicine, mortality

## Abstract

**Background:**

Tropical cyclones are a recurrent, lethal hazard. Climate change, demographic, and development trends contribute to increasing hazards and vulnerability. This mapping review of articles on tropical cyclone mortality assesses geographic publication patterns, research gaps, and priorities for investigation to inform evidence-based risk reduction.

**Methods:**

A mapping review of published scientific articles on tropical cyclone-related mortality indexed in PubMed and EMBASE (English) and SINOMED and CNKI (Chinese), focusing on research approach, location, and storm information, was conducted. Results were compared with data on historical tropical cyclone disasters.

**Findings:**

A total of 150 articles were included, 116 in English and 34 in Chinese. Nine cyclones accounted for 61% of specific event analyses. The United States (US) reported 0.76% of fatalities but was studied in 51% of articles, 96% in English and four percent in Chinese. Asian nations reported 90.4% of fatalities but were studied in 39% of articles, 50% in English and 50% in Chinese. Within the US, New York, New Jersey, and Pennsylvania experienced 4.59% of US tropical cyclones but were studied in 24% of US articles. Of the 12 articles where data were collected beyond six months from impact, 11 focused on storms in the US. Climate change was mentioned in eight percent of article abstracts.

**Interpretation:**

Regions that have historically experienced high mortality from tropical cyclones have not been studied as extensively as some regions with lower mortality impacts. Long-term mortality and the implications of climate change have not been extensively studied nor discussed in most settings. Research in highly impacted settings should be prioritized.

## Introduction

Tropical cyclones, also known as hurricanes and typhoons, are among the most destructive weather events on earth. While preparedness efforts have helped reduce mortality,^1–4^ advances have been uneven and large numbers of fatalities continue to occur.^5–8^ Prediction^9–12^ and communication^13–16^ advances have not been uniformly implemented world-wide, and optimal risk reduction strategies may vary substantially depending on geographic, socioeconomic, and cultural factors. It is currently unclear how well existing research aligns with information needs.

Successful interventions such as reversal of traffic flow on highways during evacuations in the United States (US) or use of elevated concrete cyclone shelters in Bangladesh are typically developed, evaluated, and improved through a combination of research and practical knowledge of the setting in question.^1,2,17^ Information on human mortality due to cyclones can also catalyze government policies and other interventions.^18–20^ A geographically and culturally diverse global research base is thus essential to support timely, situationally appropriate decision making.

Geographically diverse research is also necessary because climate change, demographic, and development trends may contribute to increasing hazards and vulnerability, and optimal interventions to address these issues vary widely across the globe. Warming, rising seas mean that tropical cyclones may exhibit more rapid intensification,^21,22^ increasing wind intensity and rainfall,^23,24^ higher risk of prolonged impacts due to stalling,^25,26^ more extreme storm surges,^27–29^ and exposure of new regions to cyclones.^24,30^ Many affected nations expect substantial population growth;^31^ one model suggests that by 2030, approximately 140 million people will be exposed to tropical cyclones annually, many in low- and middle-income countries of Asia and Africa.^32^ Migration toward coastal cities,^33–35^ settlement of floodplains and steep hillsides,^36–38^ loss of protective coastal marshes and mangroves,^3,39,40^ and reliance on engineered defenses^41–43^ may affect vulnerability. Research on the implications of each of these trends is needed to guide policy.

In addition, recent studies show that impacts from tropical cyclones can extend well beyond the date of the storm. Following Hurricane Maria (2017) in Puerto Rico, the official death toll of 64 prompted multiple studies which showed that thousands had lost their lives in the ensuing months.^6,7,19,44^ Similar mortality dynamics have been noted in other settings;^6,7,45–49^ long-term, all-cause excess mortality may differ substantially from immediate mortality figures based on cause of death. However, these effects are only identified when specifically investigated,^19^ and while uniform reporting systems have been proposed,^50–52^ analysis of mortality remains challenging.

Growing hazards related to climate change, worsening vulnerability related to demographic and development trends, and recent evidence for long-term and indirect mortality effects create an urgent need for research on tropical cyclone mortality that can inform future risk reduction efforts across a wide variety of settings. This mapping review seeks to describe the production of scientific knowledge on tropical cyclone mortality and to identify gaps or biases in the literature with regards to geography, methodology, and content.

## Methods

This study consisted of a structured mapping review of peer-reviewed scientific literature published in English or Chinese on the topic of human mortality in tropical cyclones. The majority of peer-reviewed literature is published in English,^53–55^ but publication volume in Chinese has increased rapidly.^56,57^ Structured searches were conducted in PubMed (National Center for Biotechnology Information, National Institutes of Health; Bethesda, Maryland USA [English]); EMBASE (Elsevier; Amsterdam, Netherlands [English]); SINOMED (Sino Medical Sciences Technology Inc.; Tianjin, China [Chinese]); and CNKI (Beijing, China [Chinese]). Searches consisted of (mortality OR death) AND (hurricane OR typhoon OR cyclone OR tropical storm OR natural disaster) in English and (死亡) AND (飓风 OR 台风 OR 气旋 OR 热带风暴 OR 自然灾害) in Chinese. Results were indexed and duplicates removed. Each article was reviewed by two separate native speakers of the language of original publication. Articles were included if they had a title and abstract available in English or Chinese, were published in or after 1985, studied tropical cyclones, hurricanes, or typhoons, and addressed human mortality as a quantitative endpoint or thematic topic. Studies exclusively presenting statistical techniques were excluded (Table 1).

**Table 1.** Article Inclusion and Exclusion Criteria

Inclusion Criteria	Exclusion Criteria
Published in English or Chinese	Published in languages other than English or Chinese
Focus on tropical cyclones, hurricanes, or typhoons	Exclusive focus on statistical techniques
Address human mortality as either a quantitative endpoint or thematic topic	Non-human mortality
Abstract available in English or Chinese	Published prior to 1985
Indexed in in PubMed, EMBASE, SINOMED, or CNKI	

Attributes were abstracted by two independent reviewers; fields included publication type, data source, data collection duration, name(s) of hurricanes studied, locations studied, mortality measurement methodology, and whether the paper referenced climate change in the abstract (a replicable proxy for whether climate change featured prominently). Results were supplemented with a global dataset of tropical cyclone disasters 1985-2019 from the Emergency Events Database (EM-DAT; Centre for Research on the Epidemiology of Disasters; Brussels, Belgium)^5^ and information on cyclone impacts in US states from the National Oceanic and Atmospheric Administration (NOAA; Washington, DC USA).^58^ Analysis was produced using R v3.6.0 (R Foundation for Statistical Computing; Vienna, Austria).^59^

## Results

**Figure 1.**
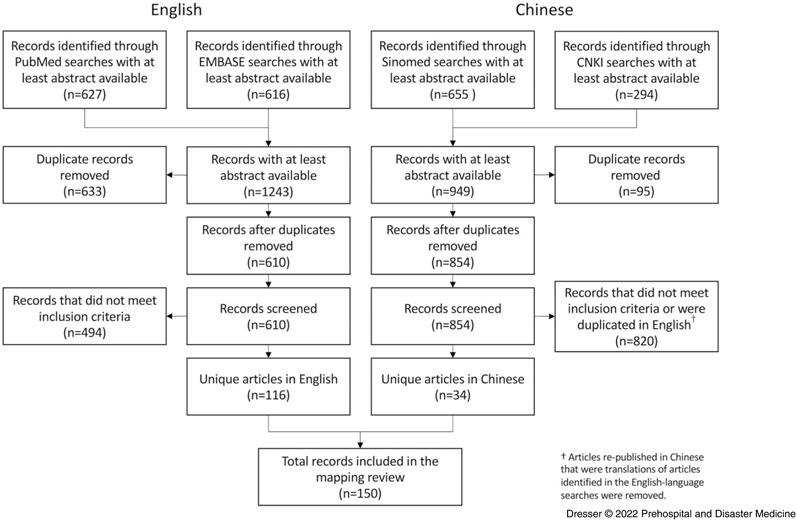
Results of Structured Process for Identification, Screening, and Inclusion of Articles for Analysis.

A total of 2,192 articles were identified in PubMed, EMBASE, SINOMED, or CNKI via structured searches. After removal of duplicates, Chinese translations of English articles, and articles that did not meet inclusion criteria (Table 1), 150 articles were retained for analysis (Figure 1).

**Figure 2.**
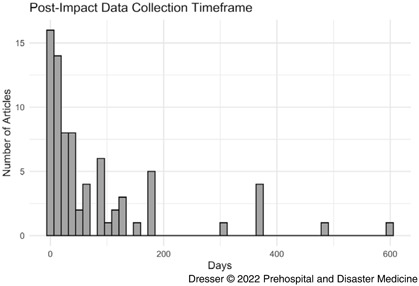
Duration of Data Collection Following Tropical Cyclone Impact in Published Studies for which Information was Available. Note: Five publications with timeframes longer than two years are not plotted.

Most articles were recent; 94 (63%) were published in 2010 or later. Original research studies accounted for 108 (72%) with other types accounting for less than 10% each (Table 2). Of 82 studies that reported a data collection timeframe, 70 (85%) collected data for six months or less after cyclone impact (Figure 2). Of the 12 studies (15%) that collected data for six months or more after storm impact, nine studied Hurricane Katrina (2005) and only one studied a location outside the US. Of the 150 studies examined, 12 (8%) referenced climate change in the abstract and 42 (28%) computed excess mortality.

**Table 2.** Attributes of Articles Included in Analysis

	Number of Articles	Percentage of Total
Total	150	100%
**Language**		
English	116	77%
Chinese	34	23%
**Year of Publication**		
1985 to 2010	56	37%
2010 to 2019	94	63%
**Publication Type**		
Research Studies	108	72%
Agency Reports	13	9%
Opinion Articles	10	7%
Review Articles	8	5%
Situation Reports	5	3%
Letters	2	1%
Case Reports	2	1%
Meta-Analyses	2	1%
**Data Source**		
Total Articles that Reported Data	123	82%
Primary Data Collection	76	62% ^a^
Pre-Existing Database or Repository	40	33% ^a^
Review or Meta-Analysis	4	3% ^a^
**Publication Content**		
Climate Change Referenced in Abstract	12	8%
Excess Mortality Calculation	42	28%

a Out of articles reporting data.

A total of 46 specific storms were analyzed individually. Some were analyzed in multiple articles and some articles discussed multiple storms; a total of 126 analyses of specific storms were identified. Of these, the top nine storms accounted for 77 analyses (61.1%) and the top 20 storms accounted for 103 (81.7%; Table 3). Twelve out of the 50 deadliest storms in EM-DAT (24%) were the subject of any studies identified in this review (Table 4).^5^

**Table 3.** Tropical Cyclones Analyzed in More than One Article and Associated Mortality,1985-2019

Name	Articles^a^	Mortality	Region
Katrina	25	1,833	Americas
Sandy	14	145	Americas
Maria ^b^	9	3,058	Americas
Rananim	7	188	Asia
Haiyan (Yolanda)	6	7,375	Asia
Ike	5	163	Americas
Andrew	4	48	Americas
Gustav	4	152	Americas
Harvey	3	88	Americas
Charley	2	15	Americas
Frances	2	49	Americas
Irma	2	105	Americas
Ivan	2	123	Americas
Ondoy (Ketsana)	2	716	Asia
Mitch	2	18,820	Americas
Nargis	2	138,375	Asia
Odisha Super Cyclone (BOB06/O5B)	2	9,843	Asia
Rammasun	2	209	Asia
Rita	2	10	Americas
Saomai	2	441	Asia
Tropical Storm One (1B) - Bay of Bengal	2	15,000	Asia
1991 Bangladesh Cyclone (Gorky/O2B)	2	138,866	Asia

Abbreviation: EMDAT, Emergency Events Database.

a Articles with substantive focus on more than one storm are listed with each storm.

b Mortality based on EMDAT and revised official death toll from Govt. of Puerto Rico.

**Table 4.** Mortality and Articles on Mortality in the 50 Deadliest Tropical Cyclones, 1985-2019

Rank	Year	Name	Mortality	Articles ^a^
1	1991	1991 Bangladesh Cyclone (Gorky/O2B)	138,866	2
2	2008	Nargis	138,375	2
3	1998	Mitch	18,820	2
4	1985	Tropical Storm One (1B) - Bay of Bengal	15,000	2
5	1999	Odisha Super Cyclone (BOB06/O5B)	9,843	2
6	2013	Haiyan (Yolanda)	7,375	6
7	1991	Thelma (Uring)	5,956	0
8	2007	Sidr	4,234	0
9	1997	Linda	3,859	0
10	2017	Maria ^b^	3,058	9
11	1998	03A	2,871	0
12	2004	Jeanne	2,782	0
13	2012	Bopha	1,901	0
14	2005	Katrina	1,833	25
15	2005	Stan	1,629	0
16	2005	Winnie	1,619	0
17	2006	Durian (Reming)	1,494	0
18	2011	Washi (Sendong)	1,439	0
19	2019	Idai	1,234	0
20	1994	Fred	1,177	0
21	1994	Gordon	1,130	0
22	1988	04B	1,074	0
23	1990	02B	957	0
24	2017	Ockhi	911	0
25	1987	Nina	882	0
26	1995	Angela	882	0
27	2008	Bilis	877	0
28	1985	Cecil	798	0
29	1989	Cecil	751	0
30	1996	O3	731	0
31	2009	Ondoy (Ketsana)	716	2
32	1996	07B	708	0
33	2009	Morakot (Kiko)	664	0
34	2008	Fengshen (Franck)	658	0
35	1993	0304-PAK (EMDAT Desig.)	609	0
36	2016	Matthew	595	1
37	1996	Frankie	585	0
38	1998	Georges	554	1
39	1989	Vera	550	0
40	2008	Hanna	537	0
41	1996	0086-BGC (EMDAT Desig.)	525	0
42	1997	0530-PER (EMDAT Desig.)	518	0
43	2009	Pepeng (Parma)	515	0
44	1990	Mike (Ruping)	503	0
45	1987	Thelma	483	0
46	1989	Gay	458	0
47	1999	02A	451	0
48	1995	Kent	445	0
49	2006	Saomai	441	3
50	1986	Wayne	435	0

Abbreviation: EMDAT, Emergency Events Database.

a Articles with substantive focus on more than one storm are listed with each storm.

b Mortality based on EMDAT and revised official death toll from Govt. of Puerto Rico.

**Figure 3.**
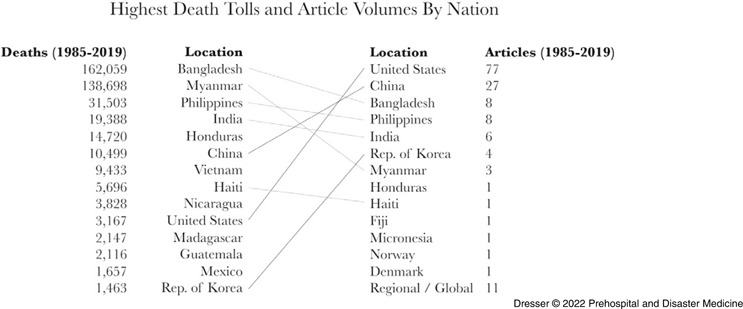
Tropical Cyclone Mortality and Article Volumes by Nation, 1985-2019.

**Figure 4.**
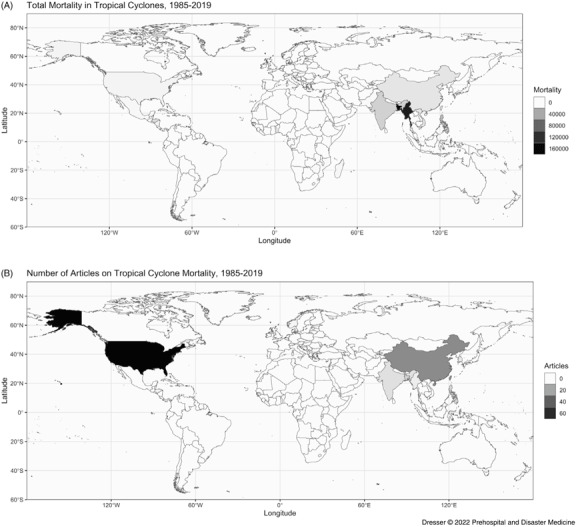
Global Distribution of **(A)** Mortality Attributed to Tropical Cyclones and Global Distribution of **(B)** Articles Analyzing Tropical Cyclone Mortality, 1985-2019.

The number of articles studying tropical cyclone mortality varied by storm impact location and are presented with cyclone mortality from EM-DAT (1985-2019) for context (Figure 3 and Figure 4). The US reported 3,167 fatalities (0.76% of global mortality) during this period^5^ but was the subject of 77 published articles (51%), 74 (96%) in English and three (4%) in Chinese. China reported 10,489 fatalities (2.51% of global mortality) and was the subject of 27 articles (18%), five (19%) in English and 22 (81%) in Chinese.^5^ Asian nations other than China reported 366,482 fatalities (87.9% of global mortality) but were the focus of 31 articles (21%), 25 (81%) in English and six (19%) in Chinese.^5^ Central American and Caribbean nations were the subject of four articles (3%) in English, though they reported 30,706 fatalities (7.36% of global mortality); inclusion of Spanish literature could alter this finding.^5^ No studies examined mortality in African nations, although 4,490 fatalities (1.07% of global mortality) were reported in this region during the study timeframe.^5^

**Figure 5.**
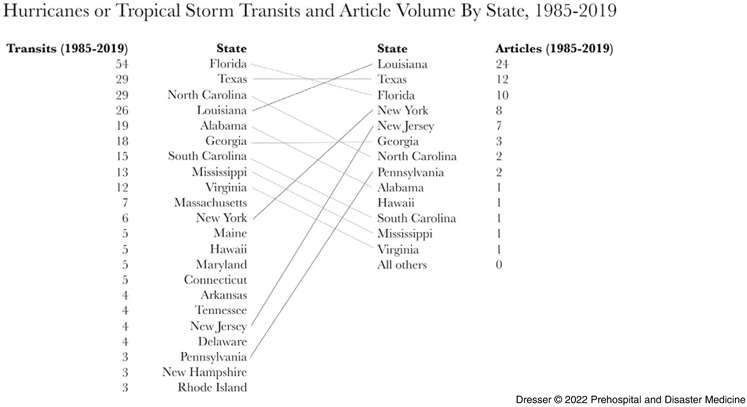
Tropical Cyclone Transits and Article Volumes by US State, 1985-2019.

Disaggregated mortality data were not available for individual US states in a uniform format;^47,60,61^ tropical cyclone transits from NOAA (1985-2019)^58^ were used to contextualize distribution of the 73 articles on specific US states (Figure 5). Louisiana, Texas, and Florida, sites of multiple recent disasters, experienced 109 cyclone transits (38.5% of the US total) and were the subject of 46 articles (63% of the US total). New York, New Jersey, and Pennsylvania experienced 13 (4.59%) cyclone transits and were the subject of 17 articles (23%), principally regarding Hurricane Sandy (2012). Other US states experienced 161 (56.9%) cyclone transits and were the topic of 10 articles (14%).

## Discussion

This review maps geography, methodology, and content for 150 scientific articles on mortality during and after tropical cyclones. While some situations have been studied in detail, for example mortality in Puerto Rico following Hurricane Maria^6,7,44^ and in sub-populations following Hurricane Sandy,^45,47,61–63^ the distribution of existing research is not proportional to historical mortality and key knowledge gaps remain.

Published articles largely focus on mortality in the US and China, which together accounted for 68% of the articles identified in this review, despite reporting less than 3.5% of recent tropical cyclone mortality.^5^ In contrast, Southeast Asia, Africa, Central America, and the Caribbean were comparatively under-represented in the literature despite high mortality. An analogous pattern was noted within the US; a disproportionate number of articles focused on states in the Northeast affected by Hurricane Sandy, while several Southern states that routinely experienced more storms were under-represented.^58^ Future research will be most useful if conducted in settings that are highly impacted by tropical cyclones and in which findings can maximally contribute to mortality prevention.

The articles identified in this review also disproportionately focus on a small number of tropical cyclones that may or may not be representative of mortality dynamics elsewhere. Nine storms accounted for 61% of analyses of specific storms identified in this study; of the 50 deadliest tropical cyclones in EM-DAT from 1985-2019, less than one-quarter were the subject of an article identified in this review. The concentration of articles on a limited sample of individual storms raises questions about the representativeness and generalizability of current knowledge.

In addition, the long-term mortality effects of tropical cyclones remain poorly understood. Only 12 studies evaluated effects more than six months after cyclone impact, and only one of these studied a location outside the US. The mechanisms proposed to mediate post-cyclone excess mortality largely involve pre-exiting medical issues, disruptions of infrastructure, and disruptions of medical care.^6,44,64–66^ It is thus plausible that the degree to which a tropical cyclone affects long-term mortality is related to factors including baseline levels of medical vulnerability, dependence on infrastructure, and infrastructure fragility in the affected area.^6,47^ As these factors vary widely on both global and national scales, it is unknown whether the long-term impacts identified in existing studies are widely generalizable or describe exceptional circumstances. Additional long-term studies are needed, particularly in settings outside the US.

Finally, few studies explicitly evaluated the implications of climate change. Most articles (92%) identified in this review did not mention climate change or related terms in the abstract, which was used as a replicable proxy for prominent consideration of this topic. Given the implications of climate change for tropical cyclone hazards,^27,35,67^ consideration of this issue is important; long-term hazard projections provide important context for the study of mortality in tropical cyclones and should be considered in risk reduction strategies.

Future years will likely witness rising seas, intensifying tropical cyclones, and worsening vulnerability in affected populations. Research on tropical cyclone mortality should prioritize lower- and middle-income settings with high historical mortality, examination of long-term effects, and evaluation of the implications of climate change. Prevention of future mortality will depend on the development of evidence-based risk reduction programs and their continuous monitoring for effectiveness during future storms. Policymakers should prioritize increased accessibility of mortality records and support for researchers working in highly affected settings.

## Limitations

This review evaluated articles published in English and Chinese; additional articles may exist in other languages and could affect results. Also, EM-DAT cyclone mortality data included a small number of extra-tropical cyclonic storms.^5^

## Conclusion

Scientific articles on tropical cyclone mortality disproportionately focus on a limited number of storms. The US and China are over-represented in the global literature relative to historical mortality, while nations in Southeast Asia, Africa, and the Americas outside the US are under-represented. Substantial knowledge gaps persist; long-term mortality effects are unclear, particularly in low-resource settings. Few publications prominently mention climate change, despite its substantial implications. Research addressing mortality related to tropical cyclones in low- and middle-income settings and over extended timeframes should be prioritized.

## References

[1] Jha A , Basu R , Basu A . Studying policy changes in disaster management in India: a tale of two cyclones. Disaster Med Public Health Prep. 2016;10(1):42–46. 26477434 10.1017/dmp.2015.116

[2] Haque U , Hashizume M , Kolivras KN , Overgaard HJ , Das B , Yamamoto T . Reduced death rates from cyclones in Bangladesh: what more needs to be done? Bull World Health Organ. 2012;90(2):150–156. 22423166 10.2471/BLT.11.088302 PMC3302549

[3] Das S , Vincent JR . Mangroves protected villages and reduced death toll during Indian super cyclone. Proc Natl Acad Sci USA. 2009;106(18):7357–7360. 19380735 10.1073/pnas.0810440106 PMC2678660

[4] Lurie N , Finne K , Worrall C , et al. Early dialysis and adverse outcomes after Hurricane Sandy. Am J Kidney Dis. 2015;66(3):507–512. 26120039 10.1053/j.ajkd.2015.04.050

[5] Guha Sapir D . EM-DAT: The Emergency Events Database - Université Catholique de Louvain (UCL). www.emdat.be. Published 2020. Accessed September 22, 2020.

[6] Kishore N , Marques D , Mahmud A , et al. Mortality in Puerto Rico after Hurricane Maria. N Engl J Med. 2018;379(2):162–170. 29809109 10.1056/NEJMsa1803972

[7] Santos-Burgoa C , Sandberg J , Suarez E , et al. Differential and persistent risk of excess mortality from Hurricane Maria in Puerto Rico: a time-series analysis. Lancet Planet Health. 2018;2(11):e478–e488. 30318387 10.1016/S2542-5196(18)30209-2

[8] Guha-Sapir D , Vogt F . Cyclone Nargis in Myanmar: lessons for public health preparedness for cyclones. Am J Disaster Med. 2009;4(5):273–278. 20014544

[9] NHC. National Hurricane Center Forecast Verification. National Hurricane Center and Central Pacific Hurricane Center. https://www.nhc.noaa.gov/verification/. Published 2017. Accessed November 22, 2020.

[10] RS. Sudden Sea: The Great Hurricane of 1938. New York USA: Little, Brown, & Co; 2004.

[11] Larson E . Isaac’s Storm: A Man, a Time, and the Deadliest Hurricane in History. New York USA: Random House; 2000.

[12] NHC. Hurricanes in History. https://www.nhc.noaa.gov/outreach/history/#new. Published 2020. Accessed June 18, 2020.

[13] WMA. SMS based Cyclone Warning System. World Meteorological Association. https://public.wmo.int/en/media/news-from-members/sms-based-cyclone-warning-system. Published 2015. Accessed June 18, 2020.

[14] Nogueira DF . Mobile-Based Early Warning Systems in Mozambique. *Uppsala University Dept of Informatics and Media Preprint.* 2019.

[15] Petrun Sayers EL , Parker AM , Ramchand R , Finucane ML , Parks V , Seelam R . Reaching vulnerable populations in the disaster-prone US Gulf Coast: communicating across the crisis lifecycle. Am J Disaster Med. 2019;14(2):121–136. 31637693 10.5055/ajdm.2019.0323

[16] AFP. Bangladesh introduces SMS cyclone alert system. https://phys.org/news/2009-06-bangladesh-sms-cyclone.html. Published 2009. Accessed June 18, 2020.

[17] EU, BW. National review of hurricane evacuation plans and policies: a comparison and contrast of state practices. Transportation Research Part A: Policy and Practice. 2003;37(3).

[18] Sills GL , Vroman ND , Wahl RE , Schwanz NT. Overview of New Orleans levee failures: lessons learned and their impact on national levee design and assessment. J Geotech Geoenviron Engineer. 2008;134(5):556–565.

[19] Arnold C. Death , statistics and a disaster zone: the struggle to count the dead after Hurricane Maria. Nature. 2019;566(7742):22–25. 30723359 10.1038/d41586-019-00442-0

[20] Stover E , Vinck P. Cyclone Nargis and the politics of relief and reconstruction aid in Burma (Myanmar). JAMA. 2008;300(6):729–731. 18698074 10.1001/jama.300.6.729

[21] Balguru K FG , Leung L . Increasing magnitude of hurricane rapid intensification in the Central and Eastern Tropical Atlantic. Geophysical Research Letters. 2018;45(9):4238–4247.

[22] Bhatia KT , Vecchi GA , Knutson TR , et al. Recent increases in tropical cyclone intensification rates. Nat Commun. 2019;10(1):635. 30733439 10.1038/s41467-019-08471-z PMC6367364

[23] Ting M , Kossin JP , Camargo SJ , Li C. Past and future hurricane intensity change along the US East Coast. Sci Rep. 2019;9(1):7795. 31127128 10.1038/s41598-019-44252-w PMC6534560

[24] Knutson T , Camargo SJ , Chan JCL , et al. Tropical cyclones and climate change assessment: Part II. Projected response to anthropogenic warming. Bull Am Meteorol Soc. 2019;101(3):303–322.

[25] Hall TM , Kossin JP. Hurricane stalling along the North American coast and implications for rainfall. npj Clim Atmos Sci. 2019;2.

[26] Kossin JP. A global slowdown of tropical-cyclone translation speed. Nature. 2018;558(7708):104–107. 29875485 10.1038/s41586-018-0158-3

[27] Marsooli R , Lin N , Emanuel K , Feng K. Climate change exacerbates hurricane flood hazards along US Atlantic and Gulf Coasts in spatially varying patterns. Nat Commun. 2019;10(1):3785. 31439853 10.1038/s41467-019-11755-z PMC6706450

[28] Oliver-Smith A. Sea Level Rise and the Vulnerability of Coastal Peoples. Bonn, Germany: United Nations University; 2009.

[29] Xiao H , Tang Y. Assessing the “superposed” effects of storm surge from a Category 3 hurricane and continuous sea-level rise on saltwater intrusion into the surficial aquifer in coastal east-central Florida (USA). Environ Sci Pollut Res Int. 2019;26(21):21882–21889. 31140083 10.1007/s11356-019-05513-3

[30] Kossin JP , Emanuel KA , Vecchi GA. The poleward migration of the location of tropical cyclone maximum intensity. Nature. 2014;509(7500):349–352. 24828193 10.1038/nature13278

[31] UN. World Population Prospects 2019. United Nations, Department of Economic and Social Affairs, Population Division. https://population.un.org/wpp/Download/Standard/Population/. Published 2020. Accessed November 20, 2020.

[32] Peduzzi P , Chatenoux B , Dao H , et al. Global trends in tropical cyclone risk. Nature Climate Change. 2012;2(4):289–294.

[33] Rigaud KK , et al. Groundswell: Preparing for Internal Climate Migration. Washington, DC USA: The World Bank; 2018.

[34] UN-DESA. World Urbanization Prospects 2018. UN Department of Economic and Social Affairs: Population Dynamics. https://population.un.org/wup/. Published 2018. Accessed November 22, 2020.

[35] Aerts JC , Botzen WJ , Emanuel K , Lin N , de Moel H , Michel-Kerjan EO . Climate adaptation. Evaluating flood resilience strategies for coastal megacities. Science. 2014;344(6183):473–475. 24786064 10.1126/science.1248222

[36] Doran KM , McCormack RP , Johns EL , et al. Emergency department visits for homelessness or inadequate housing in New York City before and after Hurricane Sandy. J Urban Health. 2016;93(2):331–344. 26979519 10.1007/s11524-016-0035-z PMC4835349

[37] Ma C , Smith T . Vulnerability of renters and low-income households to storm damage: evidence from Hurricane Maria in Puerto Rico. Am J Public Health. 2020;110(2):196–202. 31855476 10.2105/AJPH.2019.305438 PMC6951386

[38] Aune KT , Gesch D , Smith GS . A spatial analysis of climate gentrification in Orleans Parish, Louisiana post-Hurricane Katrina. Environ Res. 2020;185:109384. 32240840 10.1016/j.envres.2020.109384 PMC9045591

[39] Del Valle A , Eriksson M , Ishizawa OA , Miranda JJ. Mangroves protect coastal economic activity from hurricanes. Proc Natl Acad Sci USA. 2020;117(1):265–270. 31848234 10.1073/pnas.1911617116 PMC6955307

[40] Sun F , Carson RT . Coastal wetlands reduce property damage during tropical cyclones. Proc Natl Acad Sci USA. 2020;117(11):5719–5725. 32123063 10.1073/pnas.1915169117 PMC7084091

[41] Liu C . Shanghai Struggles to Save Itself from the Sea. In. *Scientific American*. 2011.

[42] Molinaroli E , Guerzoni S , Suman D . Do the adaptations of Venice and Miami to sea level rise offer lessons for other vulnerable coastal cities? Environ Manage. 2019;64(4):391–415. 31423556 10.1007/s00267-019-01198-z

[43] Zimmerman R , Foster S , Gonzalez JE , et al. New York City Panel on Climate Change 2019 Report Chapter 7: Resilience Strategies for Critical Infrastructures and Their Interdependencies. Ann NY Acad Sci. 2019;1439(1):174–229. 30875114 10.1111/nyas.14010

[44] Cruz-Cano R , Mead EL . Causes of excess deaths in Puerto Rico after Hurricane Maria: a time-series estimation. Am J Public Health. 2019;109(7):1050–1052. 30998411 10.2105/AJPH.2019.305015 PMC6603484

[45] Swerdel JN , Janevic TM , Cosgrove NM , Kostis JB ; Myocardial Infarction Data Acquisition System Study Group. The effect of Hurricane Sandy on cardiovascular events in New Jersey. J Am Heart Assoc. 2014;3(6):e001354. 25488295 10.1161/JAHA.114.001354 PMC4338729

[46] Toldson IA , Ray K , Hatcher SS , Louis LS . Examining the long-term racial disparities in health and economic conditions among Hurricane Katrina survivors: policy implications for Gulf Coast recovery. J Black Stud. 2011;42(3):360–378. 21905324 10.1177/0021934710372893

[47] Kim S , Kulkarni PA , Rajan M , et al. Hurricane Sandy (New Jersey): mortality rates in the following month and quarter. Am J Public Health. 2017;107(8):1304–1307. 28640678 10.2105/AJPH.2017.303826 PMC5508144

[48] Hendrickson LA , Vogt RL . Mortality of Kauai residents in the 12-month period following Hurricane Iniki. Am J Epidemiol. 1996;144(2):188–191. 8678051 10.1093/oxfordjournals.aje.a008907

[49] Dosa D , Feng Z , Hyer K , Brown LM , Thomas K , Mor V . Effects of Hurricane Katrina on nursing facility resident mortality, hospitalization, and functional decline. Disaster Med Public Health Prep. 2010;4 Suppl 1:S28–32. 23105032 10.1001/dmp.2010.11 PMC3773950

[50] Perkins GD , Jacobs IG , Nadkarni VM , et al. Cardiac arrest and cardiopulmonary resuscitation outcome reports: update of the Utstein resuscitation registry templates for out-of-hospital cardiac arrest: a statement for healthcare professionals from a task force of the International Liaison Committee on Resuscitation (American Heart Association, European Resuscitation Council, Australian and New Zealand Council on Resuscitation, Heart and Stroke Foundation of Canada, InterAmerican Heart Foundation, Resuscitation Council of Southern Africa, Resuscitation Council of Asia); and the American Heart Association Emergency Cardiovascular Care Committee and the Council on Cardiopulmonary, Critical Care, Perioperative and Resuscitation. Resuscitation. 2015;96:328–340. 25438254 10.1016/j.resuscitation.2014.11.002

[51] Task Force on Quality Control of Disaster Management, World Association for Disaster and Emergency Medicine, Nordic Society for Disaster Management. Health disaster management: guidelines for evaluation and research in the Utstein Style. Volume I. Conceptual framework of disasters. Prehosp Disaster Med. 2003;17 Suppl 3:1–177. 12938951

[52] EM, SB, HB, et al. A Framework for Assessing Mortality and Morbidity After Large-Scale Disasters. Washington, DC USA: National Academies of Science, Engineering, and Medicine; 2020. 33284570

[53] Hamel RE . The dominance of English in the international scientific periodical literature and the future of language use in science. AILA Review. 2007;20(1):53–71.

[54] van Weigen D . The language of (future) scientific communication. Research Trends. 2012(31).

[55] Amano T , Gonzalez-Varo JP , Sutherland WJ . Languages are still a major barrier to global science. PLoS Biol. 2016;14(12):e2000933. 28033326 10.1371/journal.pbio.2000933 PMC5199034

[56] QX, RF. Bigger than you thought: China’s contribution to scientific publications and its impact on the global economy. China & World Economy. 2019;27:1–27.

[57] McCarthy N . The Countries Leading the World in Scientific Publications. Statista. https://www.statista.com/chart/20347/science-and-engineering-articles-published/. Published 2019. Accessed November 24, 2020.

[58] NOAA. Historical Hurricane Tracks. https://coast.noaa.gov/hurricanes/#map=4/32/-80. Published 2020. Accessed September 5, 2020.

[59] R Development Core Team (2019). R: a language and environment for statistical computing. Vienna, Austria: R Foundation for Statistical Computing; 2019.

[60] NWS. Weather Related Fatality and Injury Statistics. National Weather Service. https://www.weather.gov/hazstat/. Published 2020. Accessed September 8, 2020.

[61] Seil K , Spira-Cohen A , Marcum J . Injury deaths related to Hurricane Sandy, New York City, 2012. Disaster Med Public Health Prep. 2016;10(3):378–385. 27074115 10.1017/dmp.2016.36

[62] Chen BC , Shawn LK , Connors NJ , et al. Carbon monoxide exposures in New York City following Hurricane Sandy in 2012. Clin Toxicol (Phila). 2013;51(9):879–885. 24059251 10.3109/15563650.2013.839030

[63] Schnall A , Law R , Heinzerling A , et al. Characterization of carbon monoxide exposure during Hurricane Sandy and subsequent Nor’easter. Disaster Med Public Health Prep. 2017;11(5):562–567. 28438227 10.1017/dmp.2016.203 PMC5708145

[64] Saulnier DD , Brolin Ribacke K , von Schreeb J . No calm after the storm: a systematic review of human health following flood and storm disasters. Prehosp Disaster Med. 2017;32(5):568–579. 28606191 10.1017/S1049023X17006574

[65] McKinney N , Houser C , Meyer-Arendt K . Direct and indirect mortality in Florida during the 2004 hurricane season. Int J Biometeorol. 2011;55(4):533–546. 20924612 10.1007/s00484-010-0370-9

[66] Burton LC , Skinner EA , Uscher-Pines L , et al. Health of Medicare Advantage plan enrollees at 1 year after Hurricane Katrina. Am J Manag Care. 2009;15(1):13–22. 19146360

[67] Bender MA , Knutson TR , Tuleya RE , et al. Modeled impact of anthropogenic warming on the frequency of intense Atlantic hurricanes. Science. 2010;327(5964):454–458. 20093471 10.1126/science.1180568

